# Surface Phenotype and Functionality of WNV Specific T Cells Differ with Age and Disease Severity

**DOI:** 10.1371/journal.pone.0015343

**Published:** 2010-12-13

**Authors:** Paolo Piazza, Curtis P. McMurtrey, Alina Lelic, Robert L. Cook, Rachel Hess, Eric Yablonsky, Luann Borowski, Mark B. Loeb, Jonathan L. Bramson, William H. Hildebrand, Charles R. Rinaldo

**Affiliations:** 1 Department of Infectious Diseases and Microbiology, University of Pittsburgh Graduate School of Public Health, Pittsburgh, Pennsylvania, United States of America; 2 Department of Microbiology and Immunology, University of Oklahoma Health Sciences Center, Oklahoma City, Oklahoma, United States of America; 3 Department of Pathology and Molecular Medicine, McMaster University, Hamilton, Ontario, Canada; 4 Department of Epidemiology and Biostatistics, University of Florida Health Sciences Center, Gainesville, Florida, United States of America; 5 Center for Research on Health Care, Pittsburgh, Pennsylvania, United States of America; Karolinska Institutet, Sweden

## Abstract

West Nile virus (WNV) infection can result in severe neuroinvasive disease, particularly in persons with advanced age. As rodent models demonstrate that T cells play an important role in limiting WNV infection, and strong T cell responses to WNV have been observed in humans, we postulated that inadequate antiviral T cell immunity was involved in neurologic sequelae and the more severe outcomes associated with age. We previously reported the discovery of six HLA-A*0201 restricted WNV peptide epitopes, with the dominant T cell targets in naturally infected individuals being SVG9 (Env) and SLF9 (NS4b). Here, memory phenotype and polyfunctional CD8^+^ T cell responses to these dominant epitopes were assessed in 40 WNV seropositive patients displaying diverse clinical symptoms. The patients' PBMC were stained with HLA-I multimers loaded with the SVG9 and SLF9 epitopes and analyzed by multicolor flow cytometry. WNV-specific CD8^+^ T cells were found in peripheral blood several months post infection. The number of WNV-specific T cells in older individuals was the same, if not greater, than in younger members of the cohort. WNV-specific T cells were predominantly monofunctional for CD107a, MIP-1β, TNFα, IL-2, or IFNγ. When CD8^+^ T cell responses were stratified by disease severity, an increased number of terminally differentiated, memory phenotype (CD45RA^+^ CD27^−^ CCR7^−^ CD57^+^) T cells were detected in patients suffering from viral neuroinvasion. In conclusion, T cells of a terminally differentiated/cytolytic profile are associated with neuroinvasion and, regardless of age, monofunctional T cells persist following infection. These data provide the first indication that particular CD8^+^ T cell phenotypes are associated with disease outcome following WNV infection.

## Introduction

West Nile virus (WNV) was first reported in the eastern United States in 1999 and has since spread westward through the entire North American continent. Reported WNV cases peaked at about 10,000 in 2003 and WNV continues to represent a considerable public health threat. Among infected individuals, almost 50% report neurologic symptoms due to encephalitis or meningitis (http://www.cdc.gov/ncidod/dvbid/westnile/surv&controlCaseCount09_detailed.htm). Furthermore, rates of WNV infection are expected to increase if global temperatures rise [1]. At this time there is no specific treatment for West Nile virus infection; intensive supportive therapy is directed toward the complications of brain infections, with anti-inflammatory medications, intravenous fluids, and intensive medical monitoring applied to severe cases. There is no prophylactic vaccine.

Most primary WNV infections are asymptomatic or exhibit a mild, flu-like illness that lasts no more than a few days. Successful resolution of primary WNV infection is associated with development of adaptive immunity to the virus. Some individuals undergo neurologic complications that can arise long after the acute phase of the infection has been resolved [2]. In addition, recent evidence suggests long term persistence of WNV, in a small but significant number of infected humans [3]. Given that MHC class I restricted CD8^+^ cytotoxic T lymphocytes (CTL) play an important role in controlling WNV infection in animal models [4], [5], [6], we reasoned that the severe CNS sequelae of WNV infection could be related to a lack of T cell immune control of the virus infection.

The objective of this study was to delineate whether CD8^+^ T cell immunity to WNV was related to neuroinvasive disease. This included assessment of memory T cell phenotypes, i.e., CD45RA (a spliced isoform of the leukocyte common antigen), CD27 (a member of the TNF receptor superfamily), CD57 (an indicator of T cells with limited proliferation potential) and CCR7 (lymph node homing receptor), and polyfunctional T cell activity, i.e., production of more than one immune mediator that are known to be involved in control of other viral infections [7], [8]. While no consensus has been reached as to what a “protective” T cell phenotype might be, a polyfunctional population of CD8^+^ T memory cells with a naïve-like phenotype (CD45RA^+^, CD27^+^, CCR7^−^) has been reported following vaccination with another flavivirus, yellow fever virus, in addition to Vaccinia and HIV infections [7], [8], [9]. Thus, we hypothesized that a lack of polyfunctional T cells with an effector memory phenotype would be related to neuroinvasive WNV infection. Our findings indicate that memory CD8^+^ T cells in persons with neuroinvasive disease were memory T cells are skewed towards a terminally differentiated, memory phenotype that was predominately monofunctional.

## Results

### Long term memory T cells specific for SVG9 and SLF9 are found at a relatively high frequency

Previously, we comparatively analyzed thousands of peptide ligands eluted from the HLA class I of infected cells, discovering six WNV-encoded, HLA-A*0201 restricted, peptide epitopes [10], [11]. Two of the six epitopes identified in these studies, SVG9 and SLF9, generated the most IFN-γ producing T cells in WNV seropositive donors. The other four epitopes generated comparatively little IFN-γ, possibly because these ligands were recognized by low frequency T cells or because these T cells do not make IFN-γ upon stimulation. To have a better understanding of the frequency of CD8^+^ T cells specific for the six HLA-A*0201 WNV peptides, we generated peptide-MHC multimers loaded with each of the peptides described (Table 1).

**Table 1:** Epitopes used to probe WNV specific immunity.

Virus	Peptide	Sequence	Location	Protein
WNV	SVG9	SVGGVFTSV	430–438	Env
WNV	RLD10	RLDDDGNFQL	78–87	NS2b
WNV	YTM9	YTMDGEYRL	518–526	NS3
WNV	SLT9	SLTSINVQA	15–23	NS4b
WNV	SLF9	SLFGQRIEV	68–76	NS4b
WNV	ATW9	ATWAENIQV	862–870	NS5
EBV	GLC9	GLCTLVAML	280–288	BMLF-1

**Figure 1:**
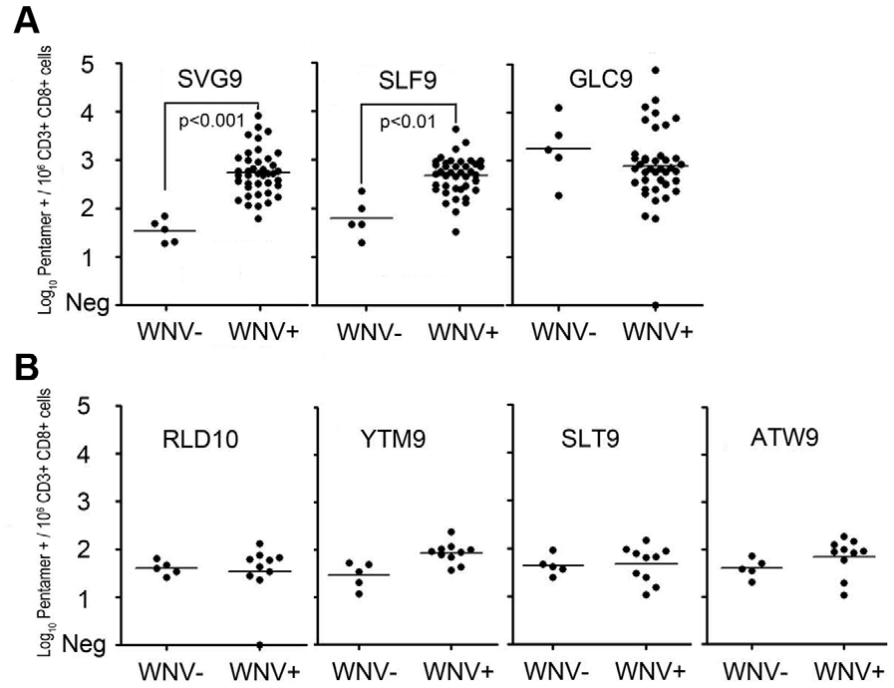
WNV p-MHC multimer positive T cells are recognized at high frequency in seropositive subjects. Log_10_ frequency of p-MHC multimer positive events per 10^6^ CD3^+^/CD8^+^ cells from WNV seropositive and WNV seronegative subjects are shown for (A) dominant epitopes and (B) non dominant WNV epitopes. All comparisons were made using a one-way ANOVA with a Tukey post-hoc test. In all cases, lines represent mean values.

Our results demonstrated that the immunodominance of SVG9 and SLF9 that we previously showed by production of IFN-γ in vitro was also evidenced by direct staining of uncultured CD8^+^ T cells with multimers specific for SVG9 and SLF9. T cells specific for these two WNV epitopes and the control, EBV epitope GLC9 were detected in the PBMC of the WNV-infected but not uninfected participants (Figure 1A). Memory T cells specific for immunodominant epitope SVG9 were recognized with a median frequency of 347/10^6^ CD3^+^ CD8^+^ cells (range, 15 - 8,529). Cells that were specific for nonstructural protein NS4b epitope SLF9 were recognized with a median frequency of 370/10^6^ CD3^+^ CD8^+^ cells (range 76 - 4,144), similar to SVG9. By comparison, the well characterized HLA-A*0201 restricted immunodominant EBV BMLF-1 (GLC9) epitope was recognized with a frequency of 467/10^6^ CD3^+^ CD8^+^ T cells (range 19 - 53,129) in the WNV positive group. Thus, after acute WNV infection, populations of peripheral memory CD8^+^ T cells specific for SVG9 and SLF9 were maintained at frequencies similar to T cells specific for an immunodominant lytic epitope of the persistent herpesvirus EBV.

In contrast to SVG9 and SLF9, the frequency of T cells specific for the four subdominant WNV epitopes (RLD10, SLT9, YTM9 and ATW9) did not differ between WNV seropositive and seronegative persons (p>0.05) (Figure 1B). These data indicate that the lack of a T cell response in the IFN-γ ELISPOT assay corresponds to a dearth of circulating T cells with T cell receptors specific for these subdominant WNV/A*0201 peptides. To confirm that T cells do recognize these four viral peptides, albeit at a frequency below the multimer staining-assay detection limit in unstimulated PBMC, T cell lines were successfully generated to all of these epitopes. Thus, memory T cells specific for subdominant WNV epitopes exist at a frequency below the detection limit of both IFN-γ ELISPOT and p-MHC staining.

### Relationship between age and WNV memory T cell frequency

**Figure 2:**
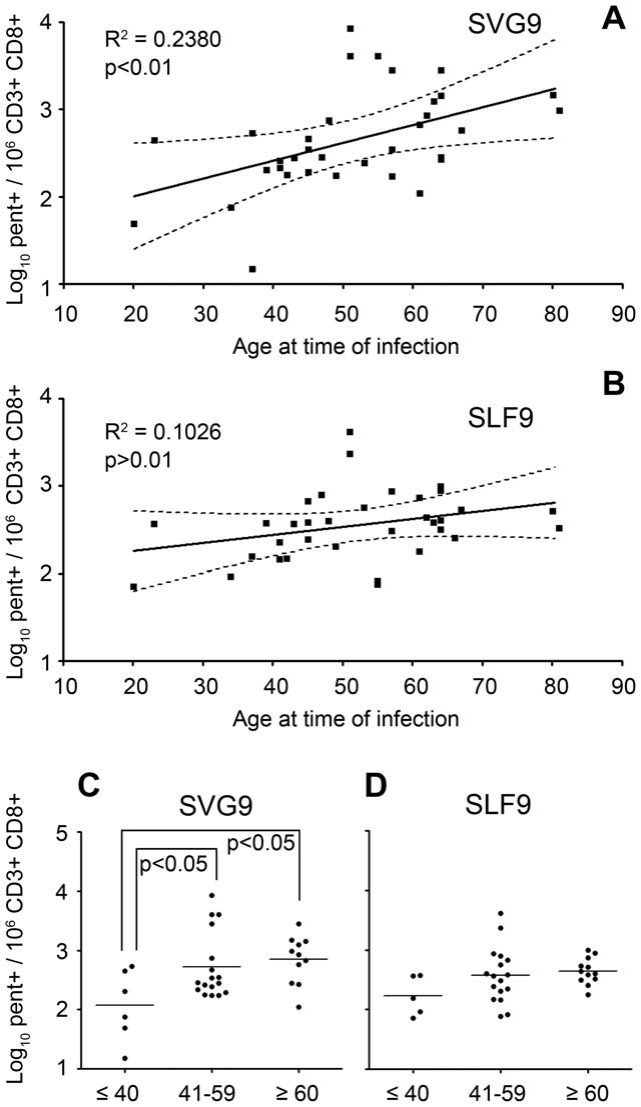
Frequency of SVG9 but not SLF9 specific cells increase with age. Log_10_ frequency of p-MHC multimer positive cells specific for SVG9 (A) and (B) SLF9 WNV epitopes vs age. The linear regression model is shown with the 95% confidence bands. Log_10_ frequency of SVG9 (C) and SLF9 (D) p-MHC multimer positive cells were divided into three age groups: subjects ≤40, 41–59, and ≥60 at time of infection. For (C) data did not fit a normal distribution so a Kruskal- Wallis ANOVA was used with Dunn's method of comparative analysis and medians are shown.

Advanced age is a risk factor for WNV disease severity [12]. We therefore examined correlations between age and SVG9/SLF9-specific T cell frequencies and found a significant positive correlation (p<0.01) of age and SVG9 p-MHC multimer-positive cell frequency (Figure 2A). When the cohort was stratified by age, there was a significant increase (p<0.05) in SVG9-specific T cells in the 41–59 and ≥60 age groups compared to subjects who were <40 years of age at the time of infection (Figure 2C). Conversely, there was no significant correlation between SLF9 p-MHC multimer positive cell frequency and age (Figure 2B) and no differences in SLF9 frequency among age groups (Figure 2D). In addition, there was no significant linear correlation with age and EBV frequency, suggesting this is WNV SVG9 specific. These data demonstrate age-related changes in WNV-specific, memory CD8^+^ T cell frequencies, and that these changes are epitope specific. Moreover, the frequency of T cells specific for the most dominant WNV/A*0201 epitope increased with age.

In contrast to the association of age with increases in T cells specific for SVG9, subjects with neuroinvasive disease did not exhibit an elevated frequency of SVG9 or SLF9 specific memory T cells (data not shown). However it should be noted that in our cohort there was not a significant age-related association with neuroinvasive disease; 14% for subjects <40 years of age and 36% for those aged 60 and over (p = 0.61). Thus, the increase in SVG9 specific T cells with age observed in our cohort was independent of disease severity.

### Long term memory surface phenotypes of p-MHC reactive cells in WNV seropositive subjects

Little information is available pertaining to the surface phenotype of long-term memory, CD8^+^ T cells to WNV. A recent study showed that equal numbers of CD45RA^+/−^ CD27^−^ (effector phenotype) and CD45RA^−^ CD27^+^ (memory phenotype) WNV-specific CD8^+^ T cells are found one month after infection [13]. This study utilized antigenic stimulation, a method that changes surface phenotype. Here, we investigated pre-antigenic stimulation *ex vivo* T cell surface phenotypes using p-MHC multimers. For clarity we report four previously defined primary memory T cell subsets: T effector memory (Tem), T central memory (Tcm), T double positive effector memory (Tdpem) and T effector memory RA^+^ (TemRA). These subsets and their corresponding phenotypes are shown in Table 2. EBV epitope GLC9 from the immediate early, lytic protein BMLF1, is included as a point of reference.

**Table 2:** Memory T cells subsets and their surface phenotype.

	CD45RA	CD27	CCR7	CD57
Tcm	−	+	+	−
Tem	−	+	−	−
Tdpem	+	+	−	−
TemRA	+	−	−	+

**Figure 3:**
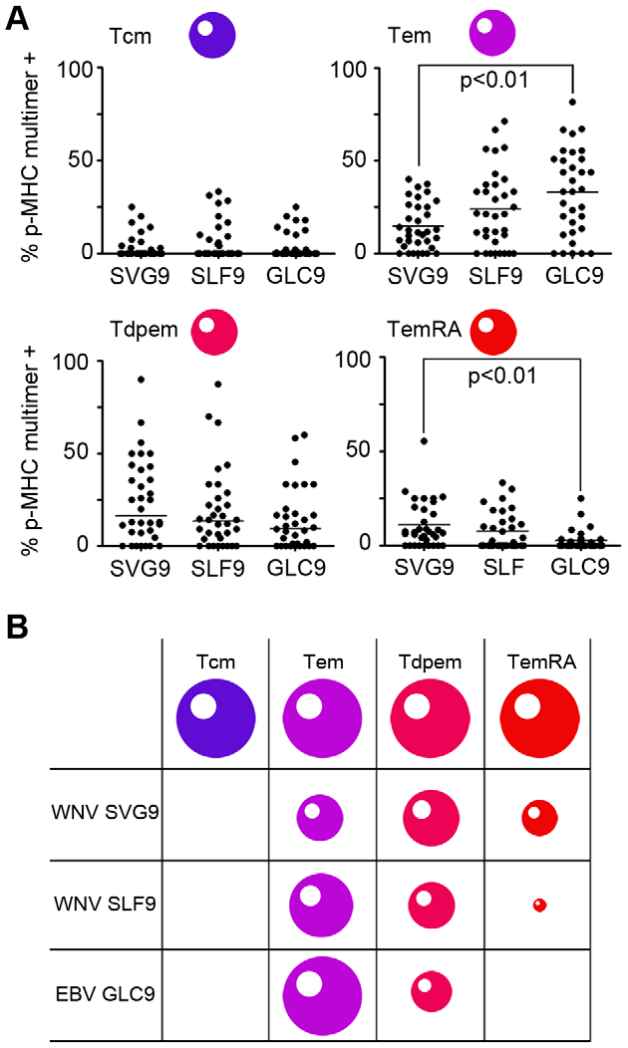
The phenotype of WNV SGV9 and SLF9 specific cells differs from that of EBV specific cells. (A) Prevalent memory phenotypes of antigen experienced T cells are shown, based on expression of CD27, CD45RA, CCR7, CD57 as defined in Table 2. For data that did not fit a normal distribution a Kruskal-Wallis ANOVA was used with Dunn's method of comparative analysis and medians are shown. In all other cases a one way ANOVA with a Tukey post-hoc test was used and means are shown. (B) The size of each cell represents the median percentage of the prevalent memory phenotypes for each of the epitopes examined in (A).

The surface phenotypes of memory T cells specific for SVG9 and SLF9 contained significant numbers of both Tem and Tdpem cells (Figure 3). For SVG9, Tdpem were most prominent, and few Tcm cells were found. An unexpected observation was that SVG9 specific T cells displaying a terminally differentiated TemRA phenotype were significantly over represented (Figure 3A, bottom right panel). This is unexpected because TemRA cells are more common during chronic infections such as human cytomegalovirus [14], while WNV causes an acute, nonpersistent infection, although this notion was challenged by recently described evidence [2], [3], [15]. In summary, T cells specific for SVG9 and SLF9 are largely Tem and Tdpem and a significant population of SVG9 specific T cells are of the TemRA phenotype (Figure 3B).

### WNV specific memory T cell phenotype differs with age and disease severity

**Figure 4:**
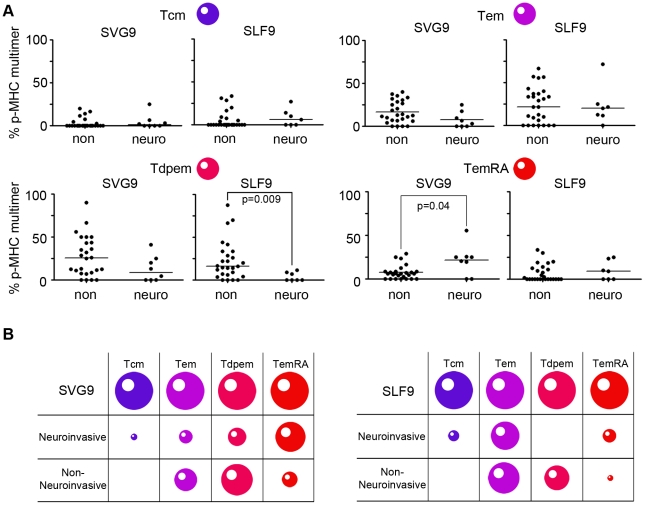
Distinct Phenotype of SGV9 and SLF9 specific cells correlate with neuroinvasive symptoms. (A) Memory phenotypes of antigen experienced T cells from donors with or without neuroinvasive symptoms are shown, based on expression of CD27, CD45RA, CCR7, CD57 as defined in Table 2. For data of a normal distribution, lines represent mean values and p values are from a student t-test analysis. For data of a non-normal distribution, lines represent median values and p values are from a Mann-Whitney test. (B) The size of each cell represents the median percentage of the prevalent memory phenotypes for each of the two clinical conditions.

Because our cohort had no age-related association with disease severity, we independently examined relationships between WNV-specific CD8^+^ T cell phenotypes, disease severity, and age. First we tested T cell phenotype profiles between subjects who had neuroinvasive disease and those who did not. For SLF9 specific cells, the Tdpem subset was overrepresented in subjects who did not develop neuroinvasive disease (Figure 4A, bottom left panel). A similar pattern was observed for SVG9, although the mean percentages were not statistically different. Interestingly, TemRA cells specific for SVG9 were more common in the neuroinvasive than in the non-neuroinvasive subjects. This increase in TemRA T cells in neuroinvasive subjects was specific to SVG9 as no difference was observed in the percentage of cells specific for subdominant epitope SLF9 (Figure 4A, bottom right panel). Finally, the Tem and Tcm median percentages were the same in both groups for SVG9 and SLF9. Epitope-specific TemRA cells are associated with WNV neuroinvasive disease while the Tdpem subset corresponds to a lack of neuroinvasive symptoms (Figure 4B).

**Figure 5:**
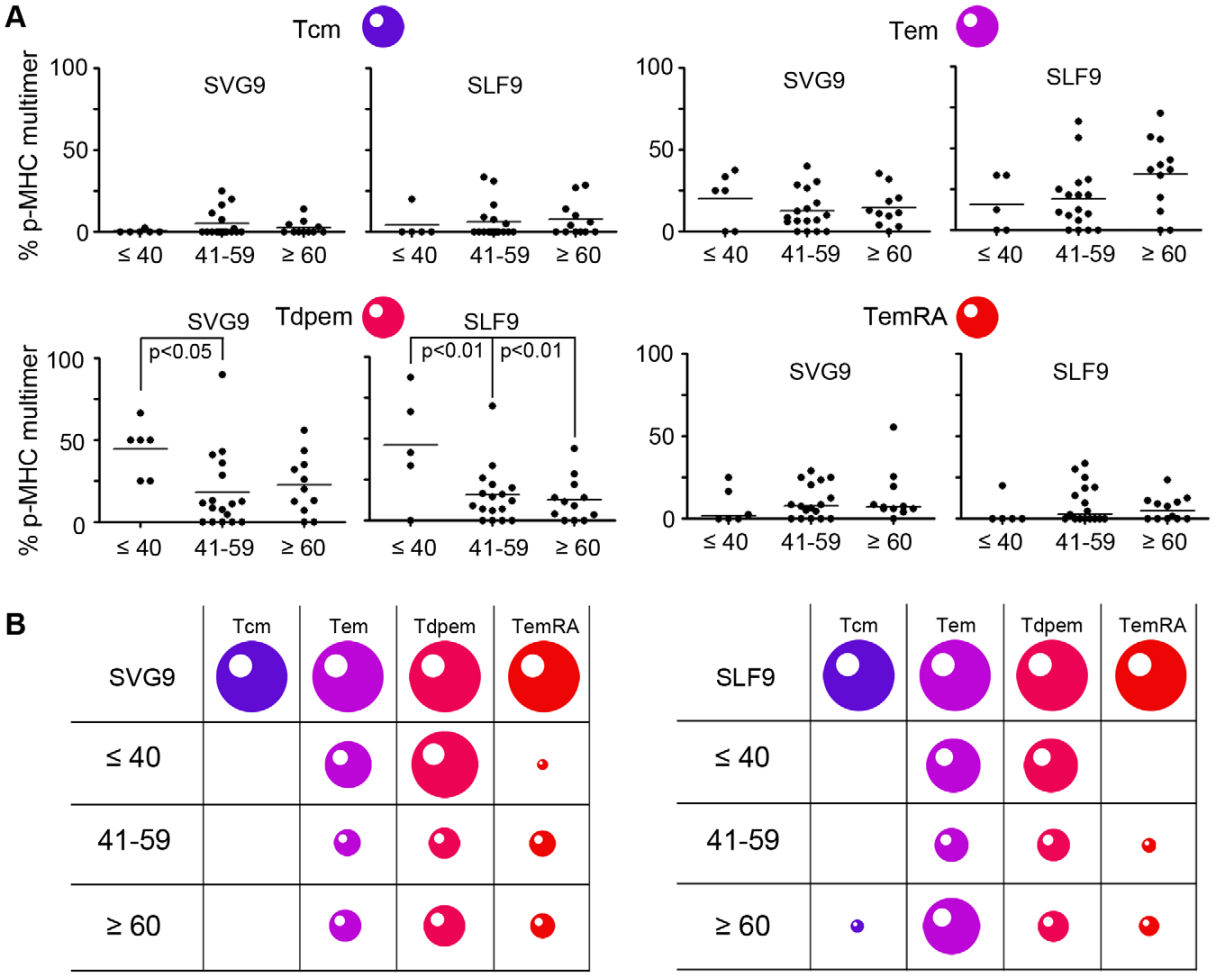
Distinct Phenotype of SGV9 and SLF9 specific cells correlate with donor age. (A) Memory phenotypes of antigen experienced T cells from donors separated by age group are shown, based on expression of CD27, CD45RA, CCR7, CD57 as defined in Table 2. For data of a normal distribution, lines represent mean values and p values are from a student t-test analysis. For data of a non-normal distribution, lines represent median values and p values are from a Mann-Whitney test. (B) The size of each cell represents the median percentage of the prevalent memory phenotypes for each age group.

Although age did not correspond to disease severity in our cohort, we nevertheless investigated CD8^+^ T cell phenotypes among defined age groups. When T cell phenotypes were stratified by age, both the SVG9 and SLF9 specific Tdpem subtype (CD45RA^+^ CD27^+^ CCR7^−^ CD57^−^) differed with age (Figure 5). The Tdpem phenotype was significantly overrepresented in subjects less than 40 years of age as compared to the older age groups (Figure 5A). It is worth noting that, as with the frequency data, there was no significant (p>0.05) gender differences in phenotype (data not shown). Together these data demonstrate that the surface phenotype of WNV memory cells vary with disease severity, age, and epitope specificity (Figure 5B).

### CD8^+^ T responses are primarily monofunctional with a high cytolytic potential in neuroinvasive disease

**Figure 6:**
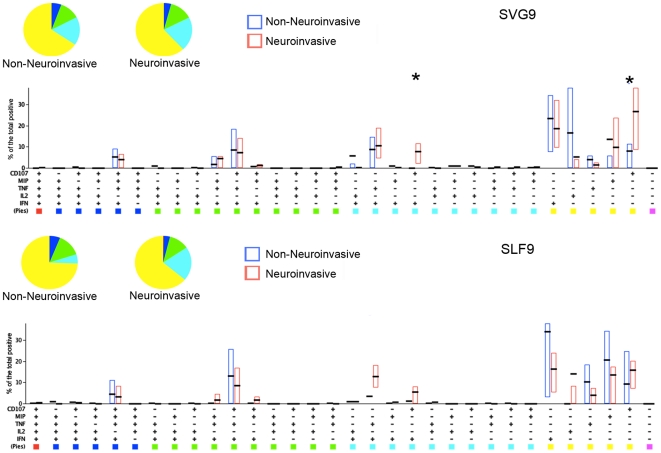
Responses of CD8^+^ cells stimulated with the SGV9 or SLF9 WNV peptide epitopes are predominantly monofunctional. PBMC from subjects with or without neuroinvasive disease were stimulated with the immunodominant SVG9 or subdominant SLF9 WNV peptide epitopes and functional responses were evaluated by multi-color flow cytometry. Percentages of CD8^+^ T cells expressing the markers shown were input in the SPICE software for display. Pies represent the distribution of responses by function for each group according to the color scheme below. Bars represent the level of response for each individual of the 32 different marker combinations. Asterisks indicate significant differences (p<0.05) by Wilcoxon signed -rank test.

Having identified phenotypes unique to donors with neuroinvasive symptoms, we next investigated the functional capacity SVG9 and SLF9 specific T cells in these patients. The stimulation of PBMC with SVG9 and SLF9 generated responding T cells that were primarily monofunctional (51.1% for SVG9 and 71.0% for SLF9) (Figure 6). Simultaneous expression of all five functions was evident in the minority of donors for both the SVG9 and the SLF9 epitopes (3/10 in either cohort, data not shown). T cells were primarily monofunctional and there was no difference in the polyfunctional capacity of T cells in donors with either neuroinvasive or nonneuroinvasive symptoms.

**Figure 7:**
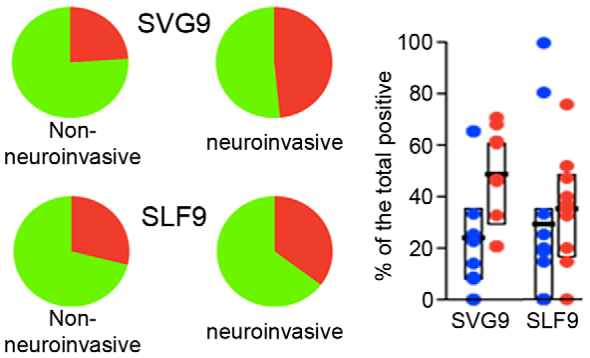
Cytolytic responses of CD8^+^ cells stimulated with the SGV9 immunodominant WNV peptide are augmented in subject with neuroinvasive involvement. PBMC from donors with or without neuroinvasive disease were stimulated with the immunodominant SVG9 or subdominant SLF9 peptide WNV epitopes and functional responses were evaluated as described. Percentages of CD8^+^ T cells expressing the cytotoxicity surrogate marker CD107a were added for each of the 32 combinations shown in Figure 5 using SPICE software. Pies represent the distribution of CD107a positive (red) and negative (green) for the two epitopes. Bars represent the interquartile range (25% –75%) of the CD107a response for the two cohorts. Wilcoxon signed-rank test was performed for the two antigens.

The neuroinvasive cohort did demonstrate a significant increase in the cytolytic associated marker CD107a (Figure 6 top panel). Again, WNV epitope specificity revealed itself as SVG9 but not SLF9 specific T cells exhibited an augmented expression of CD107a (Figure 7). This CD107a association with disease progression is consistent with the TemRA surface phenotyping data; SVG9 specific TemRA were overrepresented in neuroinvasive donors and produced CD107a upon antigenic stimulation.

## Discussion

Understanding the phenotype and functional status of T cells during a successful immune response, as well as during disease progression, is needed for the design of anti-viral immune interventions. The characterization of T cells following vaccination (YFV, vacccinia) or during chronic viral infection (HIV, EBV) indicates that particular T cell phenotypes and functional status mediate infection, yet T cell responsiveness varies from vaccine-to-vaccine and from virus-to-virus [14]. The blueprint continues to emerge in regards to the T cell response(s) that a successful vaccine or immune therapy must elicit. As T cell responses play an important role in the resolution of WNV infections, in this study we characterized WNV-specific memory CD8^+^ T cells in patients that resolve their infection, in patients with neuroinvasive disease, and stratified by age. Our data show that disease severity, age, and epitope specificity are associated with changes in memory T cell phenotype and frequency.

Our most immediate objective was to examine T cell frequencies: Immune senescence is receiving considerable attention as a risk factor during acute WNV infections in that increasing age corresponds to greater disease severity [12]. During influenza virus infections [16], [17] the frequency of virus-specific T cells decreases with age, and we posited that this would occur with WNV as well. Such was not the case, and the frequency of T cells specific for the immunodominant SVG9 epitope actually *increased* with age. These data demonstrate that CD8^+^ T cell immunity to WNV can increase as one grows older and that a dearth of WNV specific CTL mediated immunity does not correspond to disease progression.

Although T cell frequencies for the dominant WNV/A*0201 epitope increased with age, a previous study [11] found that the number of IFN-γ secreting T cells specific for SVG9 & SLF9 did not increase as patients aged. The data presented here resolve this discrepancy between total T cell numbers and IFN-γ secretion. First, the ICS data presented here reiterate that the frequency of IFN-γ producing SVG9 memory T cells does not increase with age (Figure S3A and B). However, when the activation markers CD107a, TNFα, IL2, and MIP1β are factored into the analysis, the number of SVG9 responsive T cells significantly increases with age (Figure S3C and D). These data demonstrate that the number of T cells responding to the immunodominant SVG9 epitope increase with age and that these T cells are functionally diverse.

Disease outcome was found to be independent of T cell frequency, and the next logical step was to characterize the phenotype of responding T cells. Four T cell categories were tested (Tdpem, TemRA, Tcm, Tem), and the largest proportion of WNV specific T cells exhibited a Tdpem phenotype. This memory CD8^+^ T cell subset was recently identified as the predominant subset following vaccination with YFV17D [7], suggesting that Tdpem correspond with protection from flaviviral infection. In addition to the Tdpem phenotype, Tem cells were observed for both SVG9 and SLF9, and for the SVG9 epitope a substantial number of TemRA cells were identified following infection. The Tdpem memory T cell surface phenotype is consistent with that of YFV17D vaccination and may represent a paradigm of protection for flaviviral infections.

Neuroinvasive diseases including encephalitis, meningitis and meningoencephalitis, represent the most severe conditions following WNV infection, and the relationship of T cell phenotypes and neuroinvasive outcomes was examined. Most interesting was the observation that significantly more CD45RA was expressed on by the SVG9-specific CD8^+^ T memory cells of donors with neurological involvement. These T cells expressed CD57, the an indicator of low proliferation potential, and lacked expression of CD27, confirming their terminally differentiated TemRA phenotype [18]. This increase in the frequency of T effector memory CD45RA^+^ cells following neuroinvasive infections is the first indication that changes in T cell immunity are directly associated with WNV disease severity.

TemRA cells are most commonly observed during chronic infections where antigen persists after a primary infection with pathogens such as HIV and herpesviruses [14]. Unlike these viruses, WNV infection is classified as an acute infection where sterile immunity is achieved. However, this perception may be evolving as WNV has been shown to persist in individuals with neurologic symptoms and given the observation that symptomatic WNV infections result in long term chronic symptoms. In addition, WNV RNA has been isolated from the urine of convalescent patients years after the primary infection [3]. TemRA cells may therefore indicate that WNV antigen remains following infection. Alternatively, an elevation in CD57 expressing TemRA cells in those with neuroinvasive disease may be a vestige of a strong proliferative response during the primary acute infection. Longitudinal studies that assess changes in virus-specific immunity and that test for residual virus will help to elucidate the cause and effect relationship of neuroinvation and TemRA cells.

Tdpem cells that follow YFV vaccination have a polyfunctional phenotype in that each cell produces >4 different immune mediators in addition to degranulation [7], [8]. Polyfunctional T cells have also been shown to be advantageous for the control of HIV, and we next characterized the functional status of WNV-specific T cells. We found that the majority of infected donors have WNV specific T cells that are monofunctional. In comparison to T cell functionality following vaccination with the YFV vaccine, differences in polyfunctional responses could be due to a difference in natural infection with wild-type WNV as compared to vaccination with live attenuated 17D YFV. Alternatively, differences in the class I HLA/flaviviral peptide complexes tested for WNV and YFV T cell recognition might contribute to differences in the measurement of polyfunctional T cells – each of the six WNV peptides tested here behaved differently.

In summary, we used six high-confidence WNV peptide epitopes to dissect memory T cells that remain following viral infection. T cells specific for two immunodominant WNV epitopes were detectable well after the infection was resolved and the frequency of these WNV specific T cells remained unchanged and, in some cases, increased with age; age and disease severity do not coincide with a dearth of immune reactivity. Surface phenotyping demonstrates that the majority of memory T cells have a Tdpem phenotype consistent with YFV17D vaccination, although T cells that respond to a natural WNV infection are predominatly monofuntional. In regards to neuroinvasive pathologies, T cells with a terminally differentiated phenotype represent a post-infection marker for neuroinvasive pathologies, implicating persistence of WNV antigens. Finally, age and neuroinvasive changes associated with WNV infection were specific to the dominant SVG9/A*0201 epitope complex and did not extend to the 5 other WNV epitopes presented by HLA-A*0201. Future studies of the acute immune responses that give rise to these memory T cell subsets are needed, and these studies may require both dominant and subdominant epitopes in order to identify immune response that coincide with disease progression.

## Materials and Methods

### Ethics Statement

Peripheral blood was collected from consenting subjects diagnosed with WNV in heparinized tubes in accordance with a protocol approved by the University of Pittsburgh IRB and the Research Ethics Board at McMaster University. For all donors, written, informed consent was obtained before enrolment into the study in accordance with a protocol approved by the University of Pittsburgh IRB and the Research Board at McMaster University.

### Patients and peripheral blood mononuclear cells (PBMC) preparation

Subjects were enrolled in the study following presentation of symptoms of WNV infection at local primary care clinics and detection of serum IgM by public health laboratories. HLA genotypes were determined using DNA sequence analysis at Pure Transplant Solutions (Oklahoma City, OK). Forty subjects expressing HLA A*0201 allele were selected for phenotype screening of WNV specific memory CD8^+^ T cells, using previously defined HLA A*0201 class I WNV specific multimers (Table 1). Memory responses were measured from peripheral blood samples collected 6–7 months post symptom onset. Subject data are summarized in Table 3. Of the forty patients, 10 were diagnosed with encephalitis according to CDC guidelines. A control group of 9 WNV seronegative, HLA A*0201 subjects and 2 WNV seronegative HLA A*0201 negative subjects from Pittsburgh Central Blood Bank were also included in the study. PBMCs were isolated from the blood samples by centrifugation on Ficoll (Amersham Pharmacia) and cryopreserved in RPMI 1640 medium (Invitrogen) containing 12.5% human serum albumin (Sigma) and 10% DMSO (Invitrogen).

**Table 3:** WNV infected donors.

Subject ID	Sex	Age	Days post-diagnosis	Clinical condition
77406	M	80	211	Neuroinvasive
05201	M	39	239	Non-Neuroinvasive
77411	F	48	125	Non-Neuroinvasive
55414	M	57	222	Non-Neuroinvasive
55407	M	63	210	Non-Neuroinvasive
77425	M	66	248	Neuroinvasive
77420	M	67	257	Non-Neuroinvasive
77403	F	61	256	Non-Neuroinvasive
77416	M	61	236	Non-Neuroinvasive
77413	M	37	230	Neuroinvasive
77407	F	23	216	Non-Neuroinvasive
55410	M	51	212	Non-Neuroinvasive
77409	F	81	249	Non-Neuroinvasive
77405	M	64	226	Neuroinvasive
55413	M	51	218	Neuroinvasive
55421	M	42	201	Non-Neuroinvasive
55415	F	43	240	Non-Neuroinvasive
77319	F	41	204	Non-Neuroinvasive
77330	F	45	190	Non-Neuroinvasive
77322	F	55	221	Non-Neuroinvasive
55303	F	53	222	Non-Neuroinvasive
77316	M	45	256	Non-Neuroinvasive
77303	F	57	236	Neuroinvasive
77327	M	47	271	Non-Neuroinvasive
77324	M	39	229	Non-Neuroinvasive
77311	M	64	231	Neuroinvasive
55319	M	47	216	Non-Neuroinvasive
77312	F	45	235	Neuroinvasive
77332	F	60	>200	Neuroinvasive
77307	F	55	184	Neuroinvasive
IDSA19	F	64	552	Non-Neuroinvasive
IDSA28	M	20	>200	Non-Neuroinvasive
IDSA12	M	61	86	Non-Neuroinvasive
IDSA30	M	57	556	Non-Neuroinvasive
IDSA31	F	37	237	Non-Neuroinvasive
IDB203	M	49	280	Non-Neuroinvasive
IDS104	M	41	362	Non-Neuroinvasive
IDS123	F	34	>200	Non-Neuroinvasive
IDS41	F	62	327	Non-Neuroinvasive
IDB157	M	64	261	Non-Neuroinvasive

### p-MHC multimer staining and detection

To determine the frequency of peptide-specific T cells in PBMC, cells were thawed on the same day of the assays, let recover for 2 hours at 37°C, then treated with 100 µg/ml of DNase I (Sigma-Aldrich), and washed twice in monoclonal wash buffer (HBSS with 0.01% NaN_3_, 1% FBS). After recovery, PBMC were incubated for 10 minutes at room temperature with individual WNV specific p-MHC multimers (ProImmune). Afterwards, cells were washed and then stained for 20 min at 4°C with monoclonal antibodies directed against cell surface antigens: CD3-APC-H7 and CD8 PerCP-Cy5.5 for T cell lineage; CD57-FITC, CD45RA- PE-Cy7 (all from BD Biosciences), CCR7-PE (R and D Systems) and CD27 PE-Cy5 (Beckman-Coulter) for T cell memory phenotyping; CD4, CD14, CD16 and CD19 were used to exclude cells binding nonspecifically to the p-MHC multimers (dump channel) and were labeled either with PE-TexasRed (Beckman-Coulter) or with V-450 (BD Biosciences); APC fluorotag (ProImmune) for p-MHC multimer identification. Cells were then washed and resuspended in stabilizing fixative (BD Biosciences) and analyzed on a FACS LSRII (BD Biosciences) within 24 hours. Color compensation was set up with every assay using mouse Ig binding beads (BD Biosciences). PBMC from HLA A*0201 WNV seronegative subjects were treated as described above and used as negative controls. HLA A*0201 restricted BMLF-1 EBV multimer (GLCTLVAML) was used as positive control. At least 100,000 events were collected from each subject. Data were analyzed with DIVA software (BD).

### Gating strategy and validation of WNV specific p-MHC multimers

To identify and enumerate epitope-specific CD8^+^ cytotoxic T cells, we used peptide HLA *0201 multimeric complexes specific for six individual HLA A*0201-restricted WNV epitopes previously discovered by mass spectroscopy and validated by functional testing (Table 1) in a WNV infected cohort (Table 3) [10]. In Figure S1, the polychromatic (8 color) multiparameter gating strategy used to identify WNV specific CD8^+^ T cells is described. CD4/CD14/CD16/CD19 positive cells were excluded (dump channel) to select T cells of CD8 lineage (CD3^+^/CD8^+^). Within this population the phenotypic distribution of memory markers expressed by WNV p-MHC specific T cells (i.e. CD45RA, CD27, CCR7 and CD57) was determined. Gate positions for CD45RA, CD27, CCR7 and CD57 were set for each donor based on the phenotype distribution of pMHC multimer negative CD8+ CD3+ cells. CD8^+^ T cells specific for the different A*0201/WNV peptides were thus enumerated and characterized in PBMC of WNV seropositive donors. To account for background in subsequent analyses, the frequency of CD8^+^ cells reacting with only the APC fluorotag was subtracted from the epitope specific frequencies for each donor. Thus, donor whose p-MHC cell frequency was below the APC fluorotag background frequency (negative group) were separated from those who were above (positive group). This resulted in 34/40 (85%), 34/40 (85%) and 33/40 (82.5%) of subjects positive for SVG9, SLF9 and EBV, respectively, in the positive group. The positive group was used for subsequent phenotypic analysis. To further ensure phenotypic analysis was not affected by background we grouped the samples by donors having frequencies below 3X FMO and by donors above 3X FMO. We then compared the phenotype distributions between these two groups. We found no significant difference p>0.05 in the median percentages with any phenotype (data not shown).

### Evaluation of CD8^+^ T cell polyfunctionality

Cryopreserved PBMC from a total of 20 WNV infected donors (10 non-neuroinvasive and 10 neuroinvasive) were thawed for intracellular cytokine staining (ICS) and analyzed by flow cytometry in order to measure the expression of the following immune mediators: IFNγ, TNFα, IL-2, MIP-1β, and CD107a. Cells were rested in media overnight at 37°C and then washed. Aliquots of 10^6^cells were then co-cultured with the immunodominant WNV Env peptide (SVG9, 10 µg/ml), anti-CD107a mAb FITC (BD Bioscience), GolgiStop and GolgiPlug (BD Bioscience) in a 96-well plate. The negative control was medium only and the positive control was cells stimulated with *Staphylococcus* enterotoxin B (SEB) (5 µg/ml, Sigma). Plates were incubated at 37°C for 6 h and then stained for viability with the amine-binding dye Aqua (Invitrogen) and then washed. Cells were then simultaneously stained with anti-CD3 mAb PE-Cy7 (BD Bioscience), anti-CD8 mAb PerCP-Cy5.5 (BD Bioscience), anti-CD4 mAb, anti-CD14 and anti-CD19 (all V450 conjugated; “dump channel”; BD Pharmingen), incubated in the dark for 30 minutes at room temperature (RT), and then washed. The plates were washed with cold buffer, treated with BD FACS Permeabilizing Solution 2 (200 µl/well), incubated for 10 minutes at RT, and then washed with 200 µl/well of the cold buffer. The following mAb were then added: anti-TNFα PE-Cy7 (BD Bioscience), anti-MIP-1β PE (BD Pharmingen), anti-IL2 APC (5 µL, Biolegend), and anti-IFNγ AF700 (BD Bioscience). The plates were incubated in the dark for 30 min, washed with 150 µl/well of cold buffer, and fixed with 1% paraformaldehyde in PBS. Cells were analyzed using a LSR-II 12-color flow cytometer (BD Biosciences). Polyfunctional responses to peptide or mitogenic stimuli were subtracted from cultures incubated with medium alone and assessed using the SPICE program (Version 4.3, Mario Roederer, Vaccine Research Center, NIAID, NIH). The complete gating strategy for these samples is detailed in Figure S2.

### Statistical analysis

The frequency of antigen specific, p-MHC reactive T cells does not follow a normal istribution [19] and for this reason all subsequent statistical analysis on memory cells was performed on a normalized distribution of log transformed data. Multimer positive cells were divided by total CD3+CD8+ cells. This number was then represented as events per million (multiplied by 10e6) rather than a percent (multiplied by 100). The frequency was then log transformed and reported as log _10_ p-MHC positive cells per million CD8+ CD3+ cells. Donors with no response in any assay were removed from analysis. In all cases of regression analysis, a linear regression model was used. R squared values were considered significant when p<0.01. All data were tested for a normal distribution using the Kolmogorov-Smirnov test p>0.05. Data considered to be normally distributed were analyzed using either an unpaired t-test or a one way ANOVA with a tukey post-hoc test and these data were summarized as means. When data were not normally distributed, a Mann-Whitney or a Kruskal-Wallis ANOVA with Dunn's method was used and these data were summarized as medians. For polyfunctional responses, a Wilcoxon signed-rank test used. All data was analyzed using GraphPad Prism software v4.01.

## Supporting Information

Figure S1 **Gating strategy for the identification and characterization of WNV p-MHC multimer positive CD8 T cells.** Polychromatic (8 color) staining of HLA-A*0201 restricted p-MHC multimer WNV specific CD8 ^+^ T cells from representative subject IDSA30. The gating strategy firstly identifies “singlets” by plotting forward scatter area versus forward scatter height. The singlet gate is further depleted of events identified by CD4/CD14/CD16/CD19 (dump channel) to reduce non-specific background staining. Gated CD3^+^ events are then plotted against forward scatter to identify T cells. Following this, T cells of CD8 lineage are plotted against APC labeled multimers and finally multimer positive events are characterized for the phenotypic distribution of memory markers (CD45RA/CD57/CCR7/CD27) within the multimer positive population.
(TIF)

Figure S2 **Gating strategy for the identification and characterization of poly-functional CD8 T cells responding to WNV immunodominant peptides.** After gating in the singlet events by forward scatter (height versus width) dead cells were excluded with the use of amino binding stain Aqua, cells known to bind non specifically to p-MHC multimers were further excluded via a “dump” channel consisting of antibodies to CD4, CD14, CD16 and CD19. The resulting cells were further gated for expression of CD3 and CD8 and then CD8 cells plotted against each single function.
(TIF)

Figure S3 **Correlations with age and cytokine production.** Cells were stimulated with either SVG9 (A and C) or SLF9 (B and D) and assayed for cytokine production using intracellular cytokine staining (ICS). Log_10_ frequency of IFN-γ producing cells (A and B) or cells making any functional marker by ICS (C and D) versus age.
(TIF)
