# Supplementary figures and images for: Surface Phenotype and Functionality of WNV Specific T Cells Differ with Age and Disease Severity
Source: PLoS One. 2010 Dec 13;5(12):e15343. doi: 10.1371/journal.pone.0015343 (PMC3001480; doi:10.1371/journal.pone.0015343)

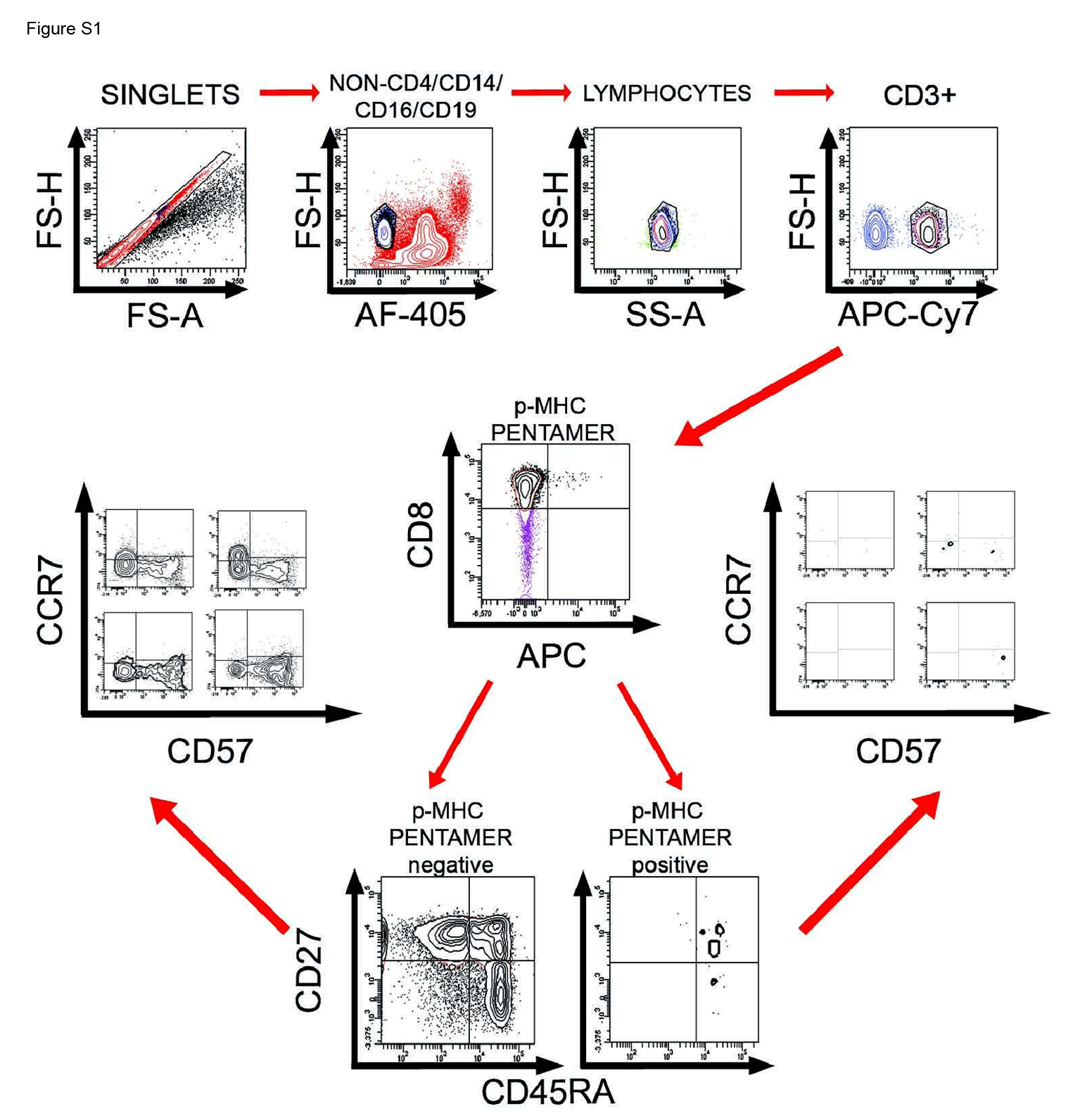

Supplement: Figure S1 — Gating strategy for the identification and characterization of WNV p-MHC multimer positive CD8 T cells. Polychromatic (8 color) staining of HLA-A*0201 restricted p-MHC multimer WNV specific CD8 + T cells from representative subject IDSA30. The gating strategy firstly identifies “singlets” by plotting forward scatter area versus forward scatter height. The singlet gate is further depleted of events identified by CD4/CD14/CD16/CD19 (dump channel) to reduce non-specific background staining. Gated CD3+ events are then plotted against forward scatter to identify T cells. Following this, T cells of CD8 lineage are plotted against APC labeled multimers and finally multimer positive events are characterized for the phenotypic distribution of memory markers (CD45RA/CD57/CCR7/CD27) within the multimer positive population. (TIF) [file pone.0015343.s001.tif]

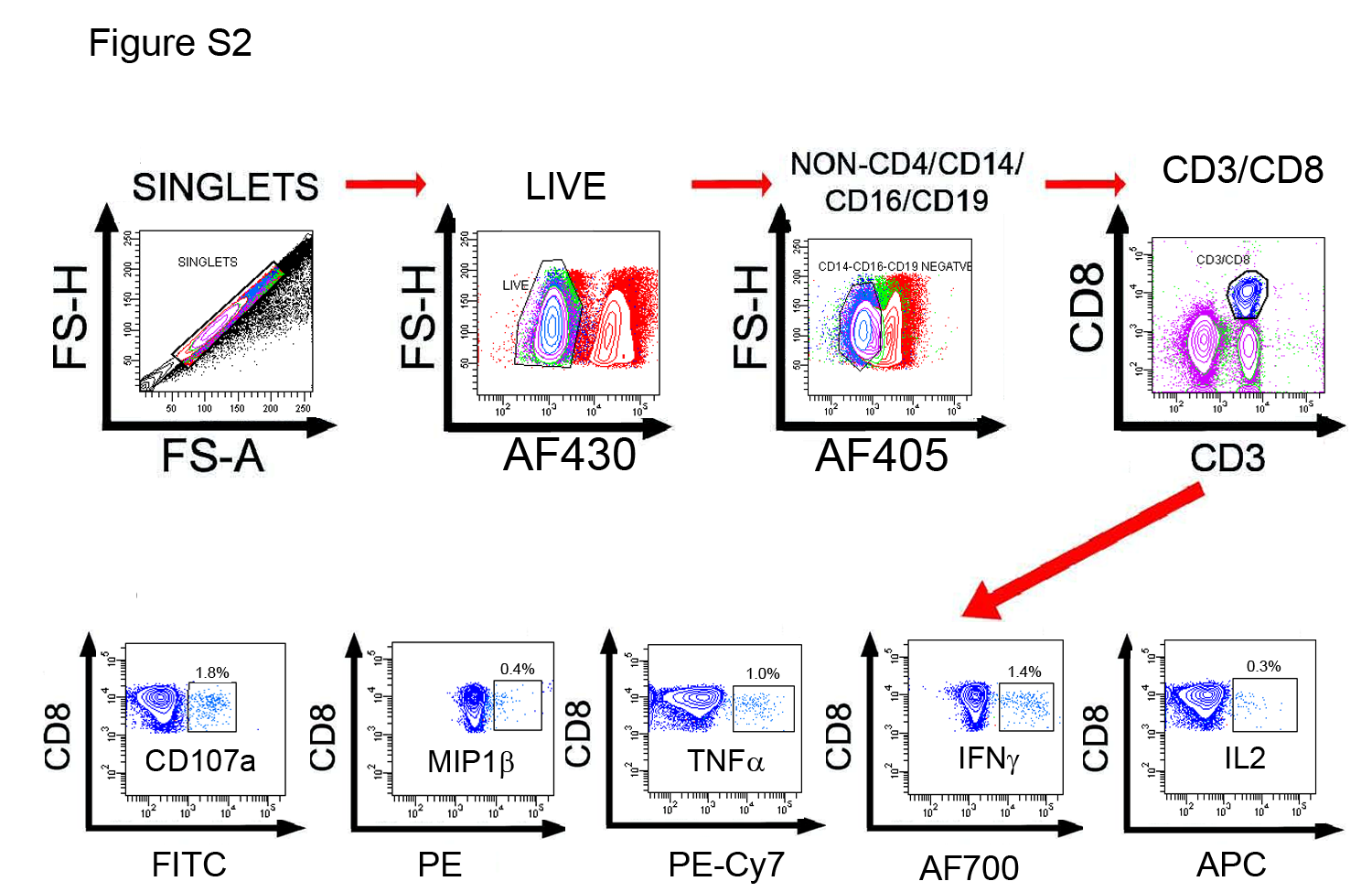

Supplement: Figure S2 — Gating strategy for the identification and characterization of poly-functional CD8 T cells responding to WNV immunodominant peptides. After gating in the singlet events by forward scatter (height versus width) dead cells were excluded with the use of amino binding stain Aqua, cells known to bind non specifically to p-MHC multimers were further excluded via a “dump” channel consisting of antibodies to CD4, CD14, CD16 and CD19. The resulting cells were further gated for expression of CD3 and CD8 and then CD8 cells plotted against each single function. (TIF) [file pone.0015343.s002.tif]

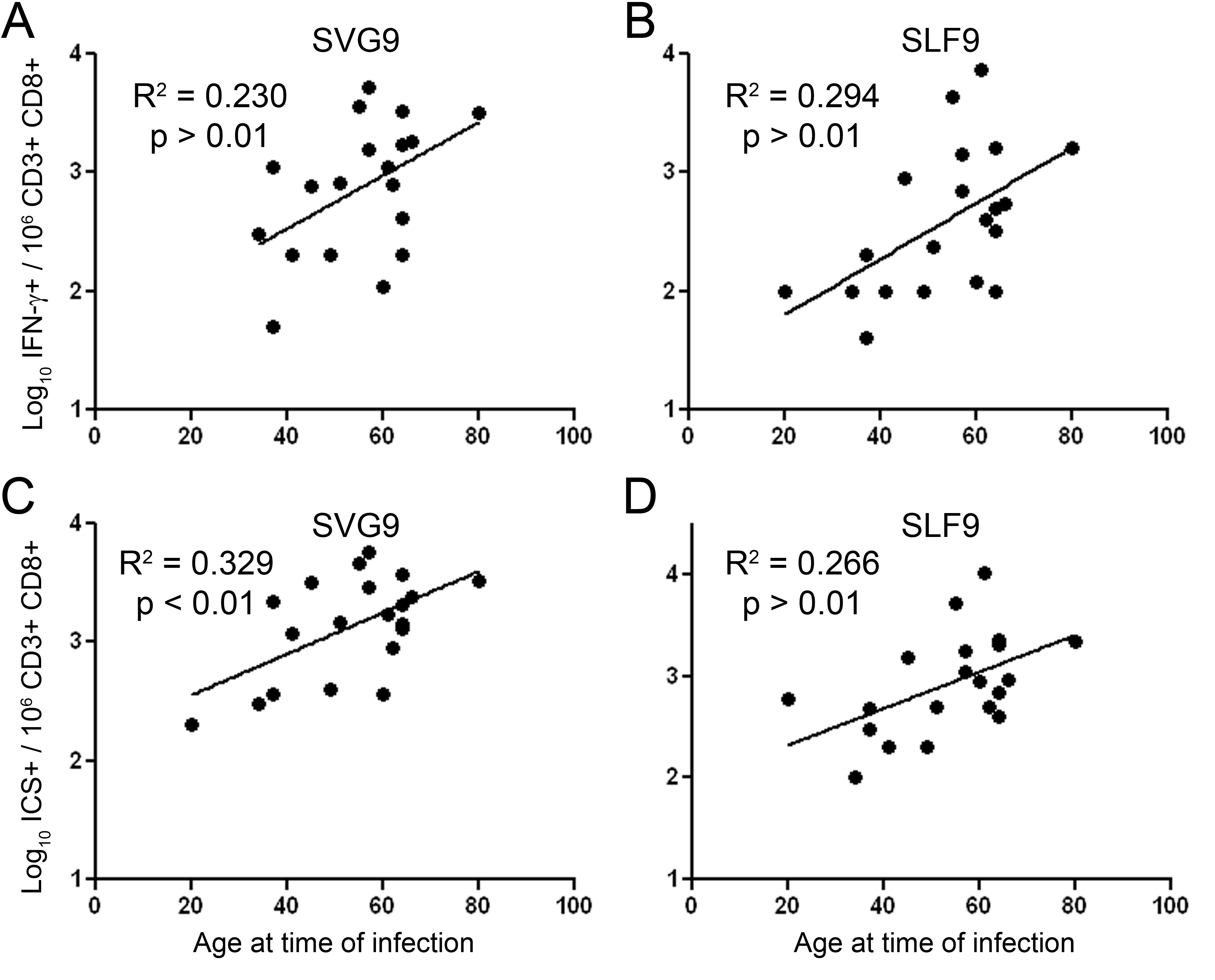

Supplement: Figure S3 — Correlations with age and cytokine production. Cells were stimulated with either SVG9 (A and C) or SLF9 (B and D) and assayed for cytokine production using intracellular cytokine staining (ICS). Log10 frequency of IFN-γ producing cells (A and B) or cells making any functional marker by ICS (C and D) versus age. (TIF) [file pone.0015343.s003.tif]
